# Outbreak of parvovirus‐B19 infection in pregnant women: Is it time to rethink a preconception or first trimester screening?

**DOI:** 10.1002/ijgo.70057

**Published:** 2025-03-05

**Authors:** Carolina Saffioti, Monica Melchio, Emilio Cristina, Caterina Costagliola, Federico Prefumo, Dario Paladini, Carolina Scala, Eddi Di Marco, Patrizia Caligiuri, Elio Castagnola, Erica Ricci

**Affiliations:** ^1^ Infectious Disease Unit IRCCS Istituto Giannina Gaslini Genoa Italy; ^2^ Department of Neurosciences, Rehabilitation, Ophthalmology, Genetics, Maternal and Child Health (DINOGMI) University of Genoa, IRCCS Istituto Giannina Gaslini Genoa Italy; ^3^ Division of Infectious Disease, Department of Health Science University of Genoa Genoa Italy; ^4^ Obstetrics and Gynecology Unit IRCCS Istituto Giannina Gaslini Genoa Italy; ^5^ Fetal Medicine and Surgery Unit IRCCS Istituto Giannina Gaslini Genoa Italy; ^6^ Molecular Diagnostics, Analysis Laboratory IRCCS Istituto Giannina Gaslini Genoa Italy

**Keywords:** fetal anemia, fetal hydrope, parvovirus B19, serological screening

Over recent months, we have seen an outbreak of parvovirus B‐19 (B19V) infections in our region, affecting pregnant women and leading to increased consultations at our regional pregnancy infection center. B19V poses significant risks during pregnancy, potentially leading to fetal anemia or hydrops, with an estimated mortality up to 29%. Nearly half of infections during pregnancy are asymptomatic and the earlier the infection, the greater the risk to the foetus.^1^ European data indicate that 40% of pregnant women are seronegative, with an estimated seroconversion rate during pregnancy ranging from 1% to 15%, with fetal transmission rates of 17%–33%.^2^, ^3^, ^4^

Routine screening for B19V during pregnancy is not recommended, while symptomatic or exposed women should undergo serology or PCR testing. Infected pregnant women require close ultrasound monitoring. This report describes the outbreak management in our center.

We collected data from all pregnant women evaluated for suspected B19V infection at the Ligurian referral center for infection in pregnancy of the Gaslini Children's Hospital in Genoa, Italy. Additionally, we gathered data from women who underwent serological or virological testing for B19V in our laboratory between January and August 2024.

DNA extracted from blood specimens was analyzed using quantitative real‐time polymerase chain reaction (PCR) for parvovirus B19, employing the Parvovirus B19VR‐GENE Kit (Argene Biomerieux, Bagno a Ripoli (FI) Italy). Simultaneously, the titration of anti‐parvovirus B19 immunoglobulin G (IgG) and IgM antibodies was performed on collected samples using an enzyme‐linked immunosorbent assay (ELISA) with the Chorus Parvovirus B19 IgG and IgM Kit (Diesse, Monteriggioni (SI) Italy).

Fifty‐seven pregnant women (median age 35.5 years) were evaluated at our center for suspected B19V infection. Of these, 41 (72%) presented for previous contact with a risk source (such as a diagnosis of erythema infectiosum in their child or professional exposure). Serological testing and viral DNA detection via real‐time PCR were conducted. Most evaluations (98%) were conducted between March and August, with the highest number of confirmed cases recorded in May (32%).

In all, 15 women (26%) had previous immunity, 8 (14%) were susceptible, while 34 (60%) had a recent infection, confirmed by positive PCR. Characteristics of infected women are described in Table 1. Notably, in four cases, serology was negative or inconclusive for diagnosis. In all cases of documented maternal infection, close follow‐up was initiated for ultrasound monitoring of potential complications. Specifically, the flow velocity of the fetal middle cerebral artery was monitored as a proxy for fetal anemia. In cases where alterations were detected, follow‐up was intensified. To date, all women have completed a 12‐week follow‐up: fetal anemia was detected in 5 (15%) cases, two of which were associated with fetal hydrops; four were treated with intrauterine transfusion and all subsequently improved. In one asymptomatic woman, the diagnosis was not suspected until a routine ultrasound scan revealed fetal hydrops. In this case, amniocentesis was performed and B19V DNA was detected directly in the amniotic fluid; unfortunately, spontaneous abortion occurred. In two other cases presenting with fetal anemia and hydrops, B19V DNA was detected in fetal blood at the first intrauterine transfusion.

**TABLE 1:** Characteristics of infected women (*n* = 34).

Characteristics	
Age (years) (median [IQR])	35.5 (31–38)
First pregnancy (*n* [%])	1 (3)
At least one risk factor for parvovirus infection (*n* [%])	33 (97)
Gestational age at infection (weeks) (three unknown) (median [IQR])	14 + 3 (8 + 6 – 20 + 2)
Symptomatic (*n* [%])	17 (50)
Fever	11 (65)
Rash	6 (35)
Arthralgia	10 (59)
Other	2 (12)^a^
Serology	
Positive (*n* [%])	25 (73)
Undetermined (*n* [%])	2 (6)
Negative (*n* [%])	2 (6)
Not performed (*n* [%])	5 (15)
Blood viral load (one unknown) (copies/mL) (median [IQR])	9 × 10^5^ (3 × 10^5^–2 × 10^6^)
Ultrasound follow‐up completed (*n* [%])	34 (100)
Fetal complications (*n* [%])	
Anemia without hydrops	3 (9)
Anemia with hydrops	2 (6)
Umbilical cord blood transfusion	4 (12)
Fetal death	2 (6)

^a^ Two women reported atypical symptoms, including one woman with associated pericarditis.

In the previous years, very few consultations were made in our center regarding suspected B19V infection. In the post‐Sars‐Cov‐2 infection era, there has been an increase in infectious diseases, which also calls for a new approach to the management of infectious diseases in pregnant women.

More parvovirus outbreaks have been reported in other countries in 2023–2024.^5^ Italy has also been affected by this outbreak, as recently described also by Tassis et al.,^6^, ^7^ who reported a marked increase in cases among pregnant women compared with previous years. However, no increase in the rate of fetal complications among infected women was observed compared with previous years. Interestingly, they noted that by implementing universal screening for all pregnant women, it was possible to diagnose the infection in 11 asymptomatic women, who subsequently underwent ultrasound monitoring in the following weeks.^7^

Similar to what has been observed by our colleagues and based on our experience, although limited, we believe it is essential to implement B19V serologic screening for women in the preconception period or during the first trimester of pregnancy, as it enables appropriate counseling and the reinforcement of hygiene measures in susceptible women while helping to prevent unnecessary anxiety in those who are already immune.

## AUTHOR CONTRIBUTIONS

*Design*: CS, ER, MM; *planning*: ER, EDM, PC; *conduct*: CS, ER, EC, CC, DP, CS; *manuscript writing*: CS, MM; *review and editing*: ER, CS, EC, FP.

## FUNDING INFORMATION

The authors received no specific funding for this work.

## CONFLICT OF INTEREST STATEMENT

The authors have no conflicts of interest.

## Data Availability

Research data are not shared.
